# Comparison of the Effects of Flexion and Extension of the Thumb and Fingers on the Position and Cross-Sectional Area of the Median Nerve

**DOI:** 10.1371/journal.pone.0083565

**Published:** 2013-12-18

**Authors:** Yasushi Toge, Yukihide Nishimura, Jeffrey R. Basford, Takako Nogawa, Midori Yamanaka, Takeshi Nakamura, Munehito Yoshida, Akira Nagano, Fumihiro Tajima

**Affiliations:** 1 Department of Rehabilitation Medicine, Wakayama Medical University, Wakayama, Wakayama, Japan; 2 Department of Orthopedic Surgery, Wakayama Medical University, Wakayama, Wakayama, Japan; 3 Department of Orthopedic Surgery, Hamamatsu University School of Medicine, Hamamatsu, Shizuoka, Japan; 4 Department of Physical Medicine and Rehabilitation, Mayo Clinic, Rochester, Minnesota, United States of America; University of Palermo, Italy

## Abstract

**Objective:**

To assess the separate effects of thumb and finger extension/flexion on median nerve position and cross-sectional area.

**Methods:**

Ultrasonography was used to assess median nerve transverse position and cross-sectional area within the carpal tunnel at rest and its movement during volitional flexion of the individual digits of the hand. Both wrists of 165 normal subjects (11 men, 4 women, mean age, 28.6, range, 22 to 38) were studied.

**Results:**

Thumb flexion resulted in transverse movement of the median nerve in radial direction (1.2±0.6 mm), whereas flexion of the fingers produced transverse movement in ulnar direction, which was most pronounced during flexion of the index and middle fingers (3.2±0.9 and 3.1±1.0 mm, respectively). Lesser but still statistically significant movements were noted with flexion of the ring finger (2.0±0.8 mm) and little finger (1.2±0.5 mm). Flexion of the thumb or individual fingers did not change median nerve cross-sectional area (8.5±1.1 mm^2^).

**Conclusions:**

Volitional flexion of the thumb and individual fingers, particularly the index and middle fingers, produced significant transverse movement of the median nerve within the carpal tunnel but did not alter the cross-sectional area of the nerve. The importance of these findings on the understanding of the pathogenesis of the carpal tunnel syndrome and its treatment remains to be investigated.

## Introduction

The carpal tunnel is the site of the most common entrapment neuropathy [1]. As a result, the tunnel and its contents have been studied extensively. Much has been learnt but the etiology of the carpal tunnel syndrome (CTS) and the relative roles of pressure, strain, swelling, and repetitive motion remain controversial [2]–[5]. What is clear, however, is that the carpal tunnel presents a dynamic milieu. Not only do tendons and the median nerve move in conjunction with hand and finger movements [6]–[11] but that hand and finger movements can change the cross-sectional area of the nerve [12].

Movement of the structures within the carpal tunnel is a complex process. Longitudinal sliding of the tendons and median nerve with hand and finger movements is intuitively obvious and well established. In fact, previous studies showed that 90° extension of the fingers from a flexed position is associated with about 2.5 mm proximal shifting of the nerve in both normal subjects and those with CTS. More recent studies have also demonstrated transverse movement of the median nerve within the tunnel during movement of the four fingers into flexion and extension [13],[14]. However, whether movements of the individual digits of the hand are associated with movement of the nerve remain unknown at present. This may be a significant gap in our understanding of the carpal tunnel as many of the repetitive activities (such as typing) that are considered to be associated with the development of CTS involve the preferential use of selected digits. The purpose of this study was to provide an understanding of the effects of flexion and extension of the individual fingers of the hand on median nerve position in the carpel tunnel in normal subjects. Such data should serve as a background for defining the pathophysiology of CTS and help design appropriate rehabilitation program for the prevention and treatment of CTS. As a first step towards achieving this goal, we determined in this study the separate effects of movements of individual digits of the hand on the position and movement of the median nerve within the carpal tunnel.

## Materials and Methods

Ultrasonography of the carpal tunnels of both wrists was performed in a sample of 15 healthy volunteers (11 men and 4 women, mean age 28.6 years, range 22–38) who had no evidence or history of pain or dysfunction of the upper extremity. The study protocol was approved by the Human Ethics Review Committee of Wakayama Medical University, School of Medicine, and a signed consent form was obtained from each subject.

Imagining was performed in a standardized manner by the first author who is an experienced ultrasonographer using the LOGIQ 500PRO (GE Medical Systems Milwaukee, WI) device with 6–13 MHz band linear transducer. Examinations were performed with the subject sitting in a relaxed position with the elbow bent at 90° of flexion and supine forearm, while resting comfortably in a shallow water tank. Water temperature was about 30°C and ultrasonography was performed with the probe of the ultrasound machine held at the level of the wrist crease about a millimeter above the surface of the skin.

Each subject was first familiarized with the experiment. Five ultrasound images were then obtained with the hand in a relaxed position with the fingers extended and adducted. Subsequently, the subjects were asked to bend and extend the individual fingers several times at various degrees of strength up to the maximal comfortable level. Each digit was then examined individually with the free digits gently held in extension by an investigator while the subject complied with the instructions to perform the “maximal comfortable flexion possible” with their free digit for five seconds while the ultrasound images were collected. The subject was then allowed to relax and the process was repeated with the other digits in a similar manner. Five 2-dimensional images were obtained at each data collection position.

Cross-sectional width and depth of the carpel tunnel were measured by the ultrasound equipment. Imaging data were imported into a personal computer and the cross-sectional area was computed using an image processing software (Scion Image for Window 4.0.2 Beta, Scion Corporation, MD). Median nerve movement was defined as the difference in the position of the nerve when the digit under consideration was at full flexion relative to its rest position at full extension. Data reported in the text and tables represent the average of five images in terms of absolute measurements as well as when normalized to the transverse motion and cross-sectional area of the median nerve. All data were expressed as mean ±SD. Differences between groups were examined for statistical significance by one-way ANOVA, and Tukey-Kramer post-hoc test. The level of significance was set at P<0.05.

## Results

**Figure 1:**
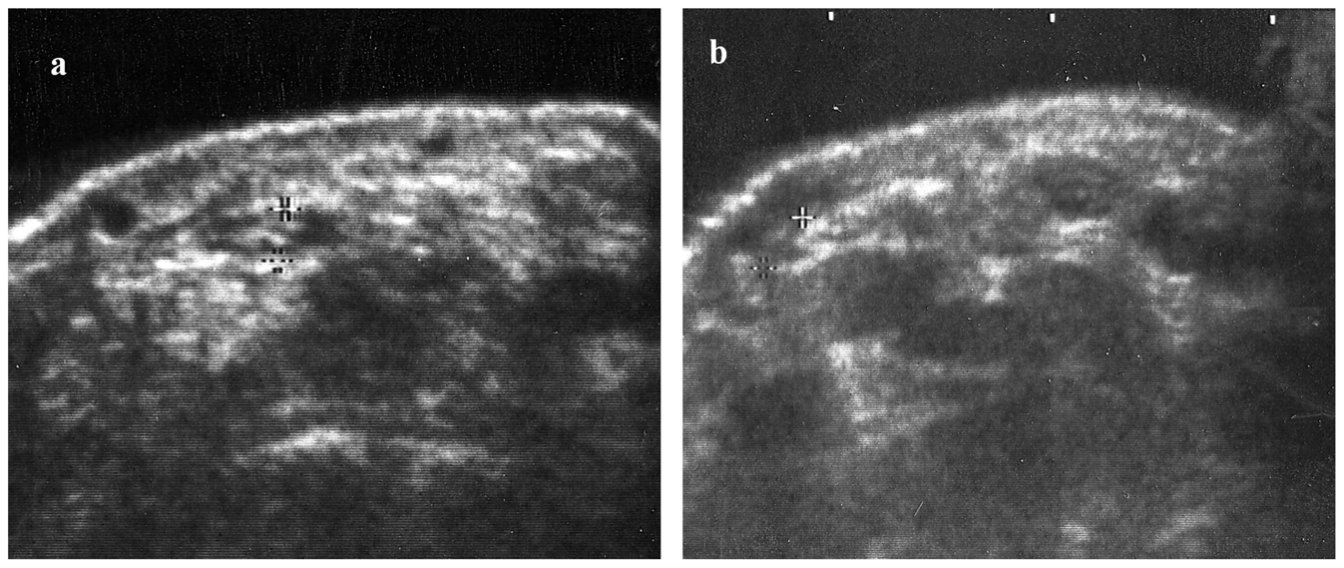
Ultrasonographic images. (a) View of the carpal tunnel with fingers at rest position. (b) View of the carpal tunnel with fingers at flexion position. Note median nerve movement in ulnar direction (from the left mark to the right) and movement of the FDS and FDP of the index and long fingers in radial direction.

The median nerve was surrounded by the flexor retinaculum, tendons of the flexor pollicis longus and the flexor digitorum superficialis (FDS) of the index and middle fingers when the hands were in resting position (i.e., with the wrist in a neutral position and the fingers and thumb extended, Fig. 1a). Table 1 shows the median nerve width, thickness and area measured at resting position.

**Table 1:** Ultrasonographic measurement of the median nerve at rest (n = 30).

Median nerve width, mm	7.0±0.8
Median nerve thickness, mm	2.2±0.3
Median nerve area, mm^2^	8.5±1.1

Values are mean±SD.

**Figure 2:**
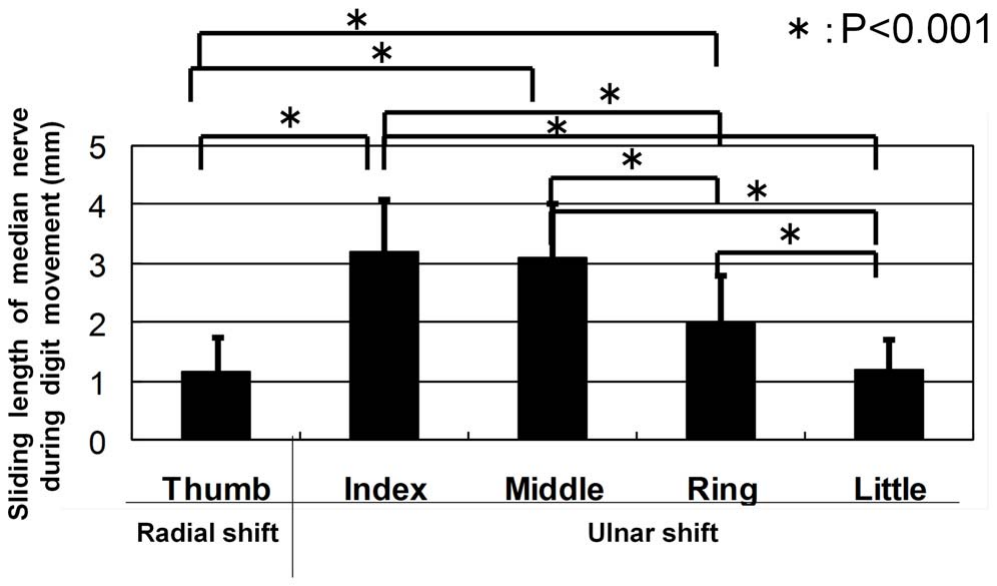
Transverse movement of the median nerve during digit movement. Data are mean±SD. *P<0.001, compared between median nerve transverse movement during flexion of each finger.

Active thumb flexion produced a radial transverse shift (1.2±0.6 mm) of the median nerve to a location just beneath the flexor retinaculum. Flexion of the fingers, however, always produced a significant ulnar transposition of the nerve that was most marked with movement of the index (3.2±0.9 mm, p<0.001) and middle fingers (3.1±1.0 mm, p<0.001) (Fig. 1b). Lesser but still statistically significant movement was noted in association with flexion of the ring finger (2.0±0.8 mm, p<0.001), while movement of the thumb and little finger produced a small but significant transverse shift of the nerve (1.2±0.6 and 1.2±0.5 mm, p<0.001, respectively) of that produced by the index and long fingers. (Fig. 2)

**Figure 3:**
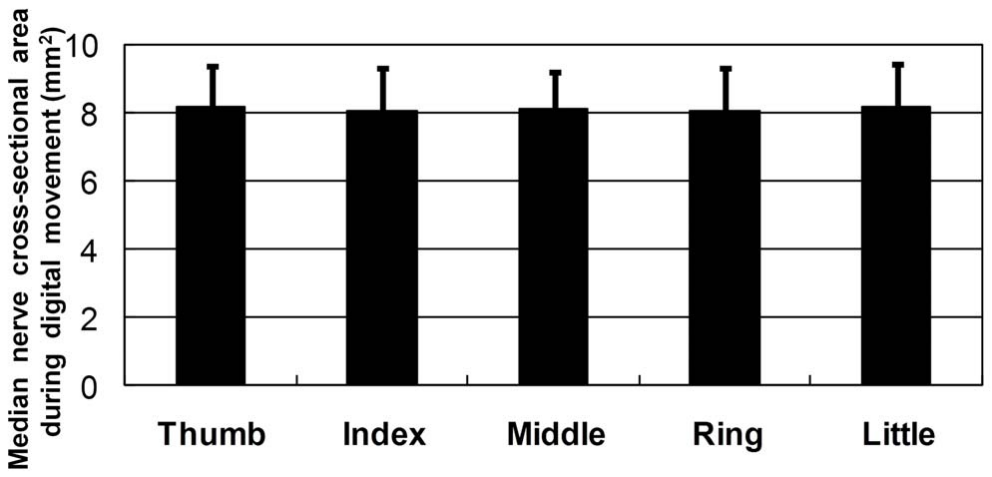
Median nerve cross-sectional area during digit movement. Data are mean±SD.

Flexion of the digits tended to produce a reduction in the cross-sectional area of the nerve but the magnitude of these changes did not reach statistical significance. (Thumb: 8.2±1.2, index 8.0±1.3, long 8.1±1.1, ring 8.1±1.2, and little 8.2±1.2 mm^2^) (Fig. 3).

## Discussion

This report extends the work of previous investigators and demonstrates that not only the median nerve is transversely displaced by mass flexion of the fingers as shown in those studies [13]–[15], but that movements of the individual digits also produce transverse motion. However, in this study, flexion of the index and long finger produced somewhat larger transverse nerve movement (about 3 mm rather than the 2 mm reported by Nakamichi and Tachibana [14]) while flexion of the other digits produced smaller movements than that recorded by the same investigators. Furthermore, the magnitude and direction of the nerve movement were dependent on the digit involved; flexion of the thumb resulted in a radial displacement of the median nerve whereas finger flexion was associated with ulnar displacement, which was most pronounced with the index and long fingers.

The relative roles of adhesive forces, tension, subcutaneous connective tissue, and potential space in the transverse movement of the median nerve with flexion of the digits may be difficult to separate. However, it is intriguing that flexion of the index and middle fingers produced the largest transverse displacement of the median nerve; in fact, the tendons of the FDS and flexor digitorum profundus are anatomically the closest to the nerve. The tendon of the flexor pollicis longus and those of the FDS and flexor digitorum profundus of the ring and little fingers are further away from the nerve and movement of these digits produced much smaller displacements of the nerve. The finding that movement of the individual digit does not alter median nerve area suggests that the forces involved in these movements are too small to produce a significant distortion of the median nerve. What are the effects of repetitive movements on the above relationship? and does the pathology underlying CTS alter the above relationship? The answers to these questions no doubt require further investigations.

A number of questions can be raised about this study. One of these is the use of ultrasonography. While an alternative technology could have been magnetic resonance imaging, we believe that ultrasonography is an appropriate and far less cumbersome and expensive choice. In fact, median nerve ultrasonography is a well-established tool in the investigation of the carpal tunnel and CTS. Indeed, there appears to be a high correlation in individuals with CTS between electrodiagnostic conduction abnormalities, self-administered assessments, and measurement of the cross-sectional area of the nerve by ultrasonography [16]. Some investigators have reported that the sensitivity and specificity of ultrasonography in the measurement of median nerve cross-sectional area and diagnosis of CTS are as high as 89% and 94.7%, respectively [17].

The study protocol is also an important issue. We used water submersion ultrasonography with 1-mm space between the probe and the wrist, as we believe that this approach avoids possible confounding effect of extrinsic pressure from the probe being applied to the volar surface of the carpal tunnel. Past reports have not always been clear about the effect of pressure of the probe on the wrist and we do not know how significant a factor this might be. The aim of such approach, however, was to avoid the issue entirely and avoid the effects of even a mild external distortion or extrinsic pressure on the positioning and movement of the carpal tunnel contents.

Splinting of the wrist and hand is a common conservative management of CTS, which is based on the premise that rest and reduced movement of the contents of the carpal tunnel facilitates resolution of inflammation or reduces irritation of its contents. The mechanism, and in fact the beneficial effects of splinting, are arguable and it is not clear whether the benefits that are observed in such patients, if any, are due to reduction in intra-tunnel pressure or reduction of movements of the contents of the tunnel [2]–[4],[18]–[20]. Our findings suggest that stabilization of the wrist and index/long fingers rather than the entire hand may be sufficient to obtain the benefits of splinting in the least restrictive manner. However, the limitation of this study is that the participating subjects were normal adults and not patients with CTS. Similar studies in such patients are warranted.

In summary, the present study indicated that flexion and extension of the fingers and thumb produced significant transverse movement of the median nerve within the carpal tunnel but had little or no acute effects on the cross-sectional area of the nerve. The significance of these findings on the etiology and treatment of CTS remain to be determined.

## References

[1] Dumitru D, Zwarts MJ (2001) Focal peripheral neuropathies. In: Dumitru D, Amato AA, Zwarts MJ (eds). Electrodiagnostic Medicine. Philadelphia, PA: Hanley and Belfus: 1043–1126.

[2] Gelberman RH, Hergenroeder PT, Hargens AR, Lundborg GN, Akeson WH (1981) The carpal tunnel syndrome-a study of carpal canal pressures. J Bone Joint Surg Am 63A: 380–383. 7204435

[3] Luchetti R, Schoenhuber R, de Cicco G, Alfarano M, Deluca S, et al (1989) Carpal-tunnel pressure. Acta Ortho Scand 60: 397–399. 10.3109/17453678909149305 2816314

[4] Lam N, Thurston A (1998) Association of obesity, gender, age, and occupation with carpal tunnel syndrome. Aust N Z J Surg 68: 190–193. 9563447 10.1111/j.1445-2197.1998.tb04743.x

[5] O'Neil BA, Forsythe ME, Stanish WD (2001) Chronic occupational repetitive strain injury. Can Fam Physician 47: 311–316. 11228032 PMC2016244

[6] Szabo RM, Bay BK, Sharkey NA, Gaut C (1994) Median nerve displacement through the carpal tunnel. J Hand Surg 19A: 901–906. 10.1016/0363-5023(94)90087-6 7876486

[7] Wilgis EF, Murphy R (1986) The significance of longitudinal excursion in peripheral nerves. Hand Clin 2: 761–766. 3025228

[8] Wright TW, Glowczewskie F, Wheeler D, Miller G, Cowin D (1996) Excursion and strain of the median nerve. J Bone Joint Surg Am 78A: 1897–1903. 10.2106/00004623-199612000-00013 8986667

[9] Dilley A, Greeting J, Lynn B, Leary R, Morris V (2001) The use of cross-correlation analysis between high-frequency ultrasound images to measure longitudinal median nerve movement. Ultrasound Med Biol 27: 1211–1218. 11597362 10.1016/s0301-5629(01)00413-6

[10] Hough AD, Moore AP, Jones MP (2000) Peripheral nerve motion measurement with spectral Doppler sonography. J Hand Surg 25B: 585–589. 10.1054/jhsb.2000.0453 11106525

[11] McLellan DL, Swash M (1976) Longitudinal sliding of the median nerve during movements of the upper limb. J Neurol Neurosurg Psychiatry 39: 566–570. 950567 10.1136/jnnp.39.6.566 PMC492349

[12] Altinok T, Baysal O, Karakas HM, Sigirici A, Alkan A, et al (2004) Ultrasonographic assessment of mild and moderate idiopathic carpal tunnel syndrome. Clin Radiol 59: 916–925. 15451352 10.1016/j.crad.2004.03.019

[13] Greening J, Lynn B, Leary R, Warren L, O'Higgins P, et al (2001) The use of ultrasound imaging to demonstrate reduced movement of the median nerve during wrist flexion in patients with non-specific arm pain. J Hand Surg 26B: 401–406. 10.1054/jhsb.2001.0582 11560418

[14] Nakamichi K, Tachibana S (1992) Transverse sliding of the median nerve beneath the flexor retinaculum. J Hand Surg 17B: 213–216. 10.1016/0266-7681(92)90092-g 1588207

[15] Nakamichi K, Tachibana S (1995) Restricted motion of the median nerve in carpal tunnel syndrome. J Hand Surg 20B: 460–464. 10.1016/s0266-7681(05)80153-6 7594983

[16] Miendany YM, Aty SA, Ashour S (2004) Ultrasonography versus nerve conduction study in patients with carpal tunnel syndrome: substantive or complementary tests? Rheumatology 43: 887–895. 15100417 10.1093/rheumatology/keh190

[17] Yesildag A, Kutluhan S, Sengul N, Koyuncuoglu HR, Oyar O, et al (2004) The role of ultrasonographic measurements of the median nerve in the diagnosis of carpal tunnel syndrome. Clin Radiol 59: 910–915. 15451351 10.1016/j.crad.2004.03.020

[18] Rempel D, Manojlovic R, Levinsohn DG, Bloom T, Gordon L (1994) The effect of wearing a flexible wrist splint on carpal tunnel pressure during repetitive hand activity. J Hand Surg 19A: 106–110. 10.1016/0363-5023(94)90231-3 8169352

[19] Luchetti R, Schoenhuber R, Alfarano M, Deluca S, de Cicco G, et al. (1994) Serial overnight recording of intracarpal canal pressure in carpal tunnel syndrome patients with and without wrist splinting. J Hand Surg. 19 B: 35–37 i. 10.1016/0266-7681(94)90045-0 8169475

[20] Gerritsen AA, de Vet HC, Scholten RJ, Bertelsmann FW, de Korm MC, et al (2002) Splinting vs surgery in the treatment of carpal tunnel syndrome: a randomized controlled trial. JAMA 288: 1245–1251. 12215131 10.1001/jama.288.10.1245

